# Retraction Note: Significance of Hall current and viscous dissipation in the bioconvection flow of couple-stress nanofluid with generalized Fourier and Fick laws

**DOI:** 10.1038/s41598-026-63326-0

**Published:** 2026-07-21

**Authors:** Muhammad Ramzan, Muhammad Javed, Sadique Rehman, Anwar Saeed, Taza Gul, Poom Kumam, Panawan Suttiarporn

**Affiliations:** 1 KMUTT Fixed Point Research Laboratory, Room SCL 802 Fixed Point Laboratory, Science Laboratory Building, Department of Mathematics, Faculty of Science, King Mongkut’s University of Technology Thonburi (KMUTT), 126 Pracha-Uthit Road, Bang Mod, Thung Khru, Bangkok, 10140, Thailand; 2 Center of Excellence in Theoretical and Computational Science (TaCS-CoE), Science Laboratory Building, Faculty of Science, King Mongkut’s University of Technology Thonburi (KMUTT), 126 Pracha-Uthit Road, Bang Mod, Thung Khru, Bangkok, 10140 Thailand; 3 Centre for Advanced Studies in Pure and Applied Mathematics, Bahauddin Zakariya University, Multan, 60800 Pakistan; 4 Department of Pure and Applied Mathematics, University of Haripur, Haripur, KPK Pakistan; 5 Cambridge Graphene Center, University of Cambridge, 9, JJ Thomson Avenue, Cambridge, CB3 0FA UK; 6 Department of Medical Research, China Medical University Hospital, China Medical University, Taichung, 40402 Taiwan; 7 Faculty of Science, Energy and Environment, King Mongkut’s University of Technology North Bangkok, Rayong Campus, Rayong, 21120 Thailand

Retraction of: *Scientific Reports* 10.1038/s41598-022-22572-8, published online 17 December 2022

The Editors have retracted this Article.

Following publication, concerns were raised regarding the VPOil Prandtl number. Specifically, Pr appears to be incorrect. Authors explained that this was a typo. However when asked to show comparison that could demonstrate that the results are correct and not derived from incorrect Pr, they provided data that show inconsistencies with the Figures reported in the Article. The Editors therefore no longer have confidence in the validity of this Article.

Poom Kumam disagrees with the retraction. Muhammad Ramzan, Muhammad Javed, Sadique Rehman, Anwar Saeed, Taza Gul, and Panawan Suttiarporn did not reply to the correspondence from the Editors about this retraction.

